# Repurposing metformin for cardioprotection: mechanisms and therapeutic potential across cardiovascular pathologies

**DOI:** 10.3389/fphar.2026.1681783

**Published:** 2026-02-02

**Authors:** Julia Khinchin, Ani Rakoubian, Valentina Romano, Thomas Ryan, Johnathan Yarbro, Satoru Kobayashi, Qiangrong Liang

**Affiliations:** Department of Biomedical Sciences, New York Institute of Technology, College of Osteopathic Medicine, Old Westbury, NY, United States

**Keywords:** AMPK, anthracycline cardiotoxicity, autophagy, cardioprotection, diabetic cardiomyopathy, heart failure, ischemia-reperfusion injury, metformin

## Abstract

Metformin, a cornerstone therapy for type 2 diabetes mellitus, has emerged as a promising cardioprotective agent with effects that extend well beyond glycemic control. This review synthesizes current evidence on the molecular and cellular mechanisms underlying metformin’s glycemic control and cardiovascular benefits, highlighting both AMPK-dependent and AMPK-independent pathways. We examine its modulation of mitochondrial function, oxidative stress, inflammation, autophagy, and apoptosis across major cardiac conditions, including ischemia/reperfusion injury, heart failure, diabetic cardiomyopathy, and anthracycline-induced cardiotoxicity. By integrating evidence from both preclinical and clinical studies, we evaluate the translational potential of metformin’s pleiotropic actions across specific cardiac pathologies and outline key directions for future research and therapeutic innovation. Together, these insights highlight metformin’s promise in reshaping cardiovascular care beyond its traditional role in diabetes management.

## 1 Metformin: origins, chemical structure, and pharmacokinetic profile

Metformin is widely recognized as the first-line oral antihyperglycemic agent for the treatment of type 2 diabetes mellitus (T2DM) globally. It is a synthetic biguanide, originally derived from a guanidine compound found in *Galega officinalis*, a medicinal plant historically used to alleviate diabetic symptoms as early as the 18th century (Dutta et al., 2023). Structurally, metformin consists of two guanidine molecules linked with the loss of ammonia, giving rise to a cationic compound under physiological conditions (Dutta et al., 2023; Jones et al., 2022).

Initially, metformin attracted limited clinical interest due to its relatively low potency and the need for high doses to achieve therapeutic effects. Earlier biguanides such as phenformin and buformin showed greater potency but were withdrawn from the market due to a high incidence of lactic acidosis (LaMoia and Shulman, 2021). This risk shifted attention toward metformin, whose milder pharmacologic profile offered a better safety margin and lower propensity for lactic acidosis.

Pharmacokinetically, metformin is absorbed primarily in the small intestine, with a bioavailability of approximately 50%–60% under fasting conditions (Jones et al., 2022). Peak plasma concentrations occur 2–3 h after oral administration, and the elimination half-life ranges from 4 to 8.7 h (Table 1), depending on the formulation (immediate- vs. extended-release). Metformin is distributed rapidly into various tissues, particularly the liver and gastrointestinal tract, and is excreted unchanged in the urine via active tubular secretion. It is not metabolized by the liver, does not undergo significant protein binding, and its clearance is highly dependent on renal function (Dutta et al., 2023; LaMoia and Shulman, 2021).

**TABLE 1:** Pharmacology and mechanistic basis of Metformin’s cardioprotective actions.

Category	Details
Pharmacokinetics	Oral bioavailability ∼50–60%; peak plasma 2–3 h; not metabolized, excreted unchanged via kidney (Dutta et al., 2023; Jones et al., 2022; LaMoia and Shulman, 2021)
Safety profile	Generally safe; main side-effects are GI-related; rare risk of lactic acidosis, esp. in renal dysfunction (F and roldi, 2024)
Core mechanisms	- AMPK activation: Key energy sensor, activated via mitochondrial inhibition (complex I) and PEN2-AXIN-LAMTOR1 complex on lysosome (Ma et al., 2022; Foretz et al., 2023; Zhang et al., 2014) - Autophagy/mitophagy: AMPK activation, mTORC1 inhibition, ULK1/TFEB modulation; context-dependent (can activate or inhibit autophagy) (Shabkhizan et al., 2025; Zamanian et al., 2024; Xiang et al., 2024; Gu et al., 2017; Xiao et al., 2017; Gao et al., 2020; Wang et al., 2018; Bhansali et al., 2020)
Redox regulation & metabolism	Inhibits mitochondrial GPD2 (affects gluconeogenesis and NADH/NAD + ratio) (Madiraju et al., 2018; Madiraju et al., 2014); decreases oxidative stress (Kamel et al., 2024; Osorio-Llanes et al., 2023)
Anti-inflammation	Inhibits NLRP3 inflammasome (Zhang et al., 2020; Yang et al., 2019), downregulates IL-6, TNF-α, and CRP, upregulates IL-10 (Almohaimeed et al., 2025; Yang et al., 2019; Schulz et al., 2024)
Other mechanistic effects	Modulates mitochondrial dynamics (e.g., MFN1, MFN2), improves mitochondrial biogenesis, inhibits apoptosis (↓ Bax/Bcl-2, ↓ caspase-3) (Wu, 2023; Ma et al., 2023)
Controversies/Complexities	Effects on autophagy/mitophagy are dose, tissue, and context dependent; AMPK-independent actions are recognized (Xu et al., 2014)

## 2 Mechanistic insights into metformin’s glucose-lowering actions

Metformin exerts pleiotropic metabolic effects extending beyond glucose control, notably regulating energy metabolism, autophagy, and redox balance. Central to these actions is AMP-activated protein kinase (AMPK), a master regulator of cellular energy homeostasis. AMPK activation by metformin depends on liver kinase B1 (LKB1) and is essential for suppressing hepatic gluconeogenesis (Shaw et al., 2005). Traditionally, this activation was attributed to inhibition of mitochondrial complex I, which raises the AMP:ATP ratio (LaMoia and Shulman, 2021; Ma et al., 2022; Foretz et al., 2023; Reczek et al., 2024). However, this occurs primarily at supra-pharmacological concentrations (millimolar), making it unlikely to explain AMPK activation at therapeutic doses (micromolar) (Di Magno et al., 2022; Fontaine, 2018; Wang et al., 2019).

**FIGURE 1:**
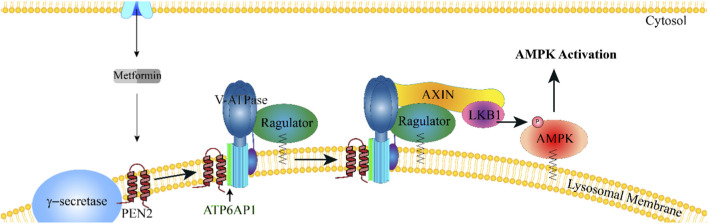
Mechanism by which low-dose Metformin activates AMPK. Clinically relevant concentrations (micromolar) of metformin directly bind to PEN2 on the lysosomal membrane, inducing a conformational change that enables PEN2 to interact with ATP6AP1, an accessory subunit of the lysosomal proton pump v-ATPase, inhibiting its activity. v-ATPase inhibition leads to a conformational change in the v-ATPase-Ragulator complex, facilitating lysosomal translocation of AXIN, which recruits and serves as a scaffold for LKB1. Once recruited to the lysosomal membrane, LKB1 phosphorylates AMPK, leading to its activation.

Recent findings identify an AMP-independent lysosomal mechanism that better accounts for metformin’s effects at clinically relevant concentrations. Through direct binding to presenilin enhancer 2 (PEN2) on the lysosomal membrane, metformin inhibits v-ATPase via interaction with ATP6AP1, triggering recruitment of the AXIN-LKB1 complex to the lysosome (Ma et al., 2022; Foretz et al., 2023). This conformational rearrangement allows LKB1 to phosphorylate AMPK locally, independently of cellular energy charge or glucose levels (Zhang et al., 2014). This PEN2-v-ATPase-Ragulator pathway (Figure 1) links metformin to lysosomal nutrient sensing rather than mitochondrial stress, offering a potentially safer mode of AMPK activation. Although unconfirmed in cardiac tissue, this mechanism may underlie metformin-induced AMPK activation in the heart (Mouli et al., 2025; Felgueiras et al., 2022; Vaez et al., 2016; Saeedi et al., 2008; Zhang et al., 2020), where v-ATPase also governs substrate utilization and is implicated in cardiac pathology (Wang et al., 2022; Chen et al., 2025).

**FIGURE 2:**
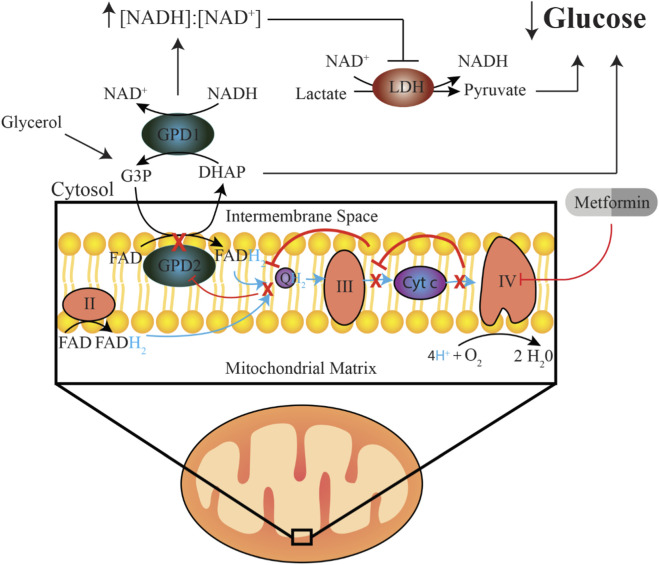
Proposed mechanism by which low-dose Metformin reduces hepatic glucose production. Clinically relevant concentrations (micromolar) of metformin inhibit complex IV of the electron transport chain. When complex IV is inhibited, electrons cannot be efficiently transferred to the final electron acceptor, oxygen, causing upstream complexes to become overwhelmed with electrons. The resulting ETC backlog inhibits the activity of GPD2, as GPD2 donates its electrons (from the reduction of FAD to FADH2) to the oxidized form of CoQ10 (ubiquinone, Q) to generate the reduced form (ubiquinol, QH_2_). Indirect inhibition of GPD2 results in reduced conversion of G3P to DHAP, resulting in reduced glycerol-derived gluconeogenesis. Further, impaired GPD2 function disrupts the glycerol-phosphate shuttle, leading to an increased cytosolic redox state (NADH:NAD), which inhibits the activity of LDH, thereby reducing lactate-derived gluconeogenesis.

While AMPK activation explains many of metformin’s systemic effects, its glucose-lowering action appears to rely more directly on redox modulation of hepatic gluconeogenesis. Metformin inhibits gluconeogenesis from redox-dependent substrates such as lactate and glycerol by targeting mitochondrial glycerol-3-phosphate dehydrogenase (mGPD2), increasing the mitochondrial NADH/NAD^+^ ratio and disrupting the glycerol-3-phosphate shuttle (Madiraju et al., 2018; Madiraju et al., 2014). Although direct inhibition of mGPD2 remains debated (Di Magno et al., 2022), metformin likely exerts secondary effects through complex IV inhibition, as shown by elevated hepatic glycerol-3-phosphate and evidence of copper-mediated complex IV modulation (Figure 2) (LaMoia et al., 2022).

Together, these redox and lysosomal pathways allow metformin to suppress hepatic glucose production without broadly impairing mitochondrial respiration. This selective mechanism is especially effective in obesity and advanced T2DM, where enhanced adipose lipolysis increases glycerol flux to the liver (Pu et al., 2020). Beyond the liver, redox modulation may also contribute to metformin’s cardioprotective effects, particularly under conditions of mitochondrial or metabolic stress (Lewis et al., 2016).

## 3 Metformin as a cardioprotective agent: mechanisms and evidence across diverse cardiac pathologies

Although widely used as a first-line therapy for type 2 diabetes, metformin exerts beneficial effects that extend well beyond glucose control. In patients with diabetes, metformin therapy is consistently associated with a lower incidence of cardiovascular events compared with sulfonylureas or insulin, even with similar glycemic control (Hong et al., 2013), although one study reported reductions primarily in all-cause rather than cardiovascular mortality (Bergmark et al., 2019). In non-diabetic individuals, particularly those with obesity or metabolic syndrome, metformin improves insulin sensitivity, lipid profiles, and body weight (Seifarth et al., 2013), thereby creating a favorable cardiometabolic milieu that may support downstream cardiovascular protection.

These benefits arise from several converging mechanisms (Table 1). Metformin activates AMPK and related cytoprotective pathways, enhances endothelial function, suppresses inflammation and oxidative stress (Luo et al., 2019; Mthembu et al., 2025; Rozier et al., 2021). It also modulates autophagy via AMPK-mTORC1 and lysosomal regulation (Shabkhizan et al., 2025; Zamanian et al., 2024; Xiang et al., 2024; Gu et al., 2017; Xiao et al., 2017; Gao et al., 2020; Wang et al., 2018; Bhansali et al., 2020; Wei et al., 2025; Lu et al., 2021). Although autophagy responses may vary by context (Huang et al., 2020; Van et al., 2023), the overall effect promotes cellular homeostasis and reduces metabolic stress.

Clinical studies in non-diabetic cohorts have demonstrated modest vascular and myocardial improvements, but findings remain variable and definitive cardiovascular outcomes trials are still lacking (Lexis et al., 2014; Preiss et al., 2016; Preiss et al., 2014; Jadhav et al., 2006; Kamel et al., 2024). Thus, despite a strong mechanistic rationale for cardioprotection, metformin’s clinical efficacy likely depends on disease context, timing of intervention, and underlying metabolic status.

The following sections examine how these core mechanisms translate into condition-specific effects across major cardiovascular pathologies, highlighting how metformin’s multifaceted actions may offer therapeutic value across diverse cardiac settings.

### 3.1 Metformin and ischemia/reperfusion injury

Ischemia/reperfusion (I/R) injury is a major contributor to cardiac dysfunction, driven by mitochondrial damage, oxidative stress, inflammation, and cell death upon restoration of blood flow. Metformin demonstrates cardioprotective effects against I/R injury through multiple mechanisms, involving both AMPK dependent and independent pathways (Osorio-Llanes et al., 2023).

#### 3.1.1 Preclinical evidence

Administration of metformin before ischemia or at reperfusion reduces myocardial injury in diabetic and non-diabetic models without altering blood glucose. During early reperfusion, metformin activates AMPK and promotes endothelial nitric oxide synthase (eNOS) phosphorylation at Ser1177(48), effects abolished in AMPK- or eNOS-deficient mice (Gundewar et al., 2009), confirming pathway dependence. In Langendorff-perfused rat hearts, metformin at reperfusion decreases infarct size, improves ventricular function, and suppresses NLRP3 inflammasome activation, caspase-1 activity, and IL-1β/IL-18 expression; these benefits disappear with AMPK inhibition (Zhang et al., 2020). At higher doses, metformin confers protection via AMPK-independent inhibition of mitochondrial complex I (Mohsin et al., 2019).

Metformin also mimics pharmacologic preconditioning (e.g., sevoflurane) by activating Akt and upregulating anti-apoptotic proteins such as Bcl-xL (Rozier et al., 2021). In models of metabolic syndrome and coronary artery disease (CAD), chronic metformin therapy reduces cardiomyocyte apoptosis and improves myocardial function under ischemic stress (Stone et al., 2024).

#### 3.1.2 Clinical evidence

Metformin reduces the risk of rehospitalization for chest pain in hyperglycemic patients with ischemia and no obstructive CAD (Mone et al., 2025). Observational data further suggest that long-term metformin use lowers the risk of myocardial infarction by approximately 33% (Holman et al., 2008). Notably, metformin use at the time of acute MI has been associated with higher early cardiovascular risk, whereas initiation after MI confers benefit in patients with type 2 diabetes (Bromage et al., 2019), indicating that the cardioprotective effects of metformin are timing dependent.

In contrast, evidence in non-diabetic populations remains inconsistent: some studies report improved vascular function and reduced myocardial ischemia in non-diabetic women treated with metformin (Jadhav et al., 2006), whereas the GIPS-III trial showed no improvement in left ventricular ejection fraction after myocardial infarction in non-diabetic patients (Lexis et al., 2014; Hartman et al., 2017), and the CAMERA trial found no significant regression of carotid intima-media thickness or reduction in vascular inflammation (Preiss et al., 2016; Preiss et al., 2014). Collectively, these findings underscore the need for more targeted and adequately powered studies in non-diabetic populations.

#### 3.1.3 Mechanistic insights

Metformin preserves mitochondrial integrity, a key determinant of I/R injury, by stabilizing membrane potential, inhibiting complex I during early reperfusion and preventing mitochondrial permeability transition pore (mPTP) opening, thus limiting ROS generation and apoptosis (Osorio-Llanes et al., 2023; Mohsin et al., 2019; Chen et al., 2024). It further enhances mitochondrial quality control by attenuating ferroptosis and activating the Nur77-IDH1 axis, thereby reducing ROS and promoting cardiomyocyte survival (Wu et al., 2025). Metformin’s AMPK-dependent actions also extend to cardiopulmonary I/R models, where it reduces oxidative stress, suppresses inflammatory cytokines (TNF-α, IL-1β), and inhibits NF-κB signaling, collectively preserving tissue integrity (Liu et al., 2024). Reports that metformin may inhibit autophagy (Huang et al., 2020) highlight the complexity of its regulation of mitochondrial homeostasis and underscore the need for further investigation.

High-dose metformin treatment to inhibit complex I during early reperfusion protects the aged mouse heart via decreased mitochondrial permeability transition pore opening.

#### 3.1.4 Summary

Collectively, metformin mitigates I/R injury through converging mechanisms including AMPK activation, mitochondrial preservation, suppression of inflammation and oxidative stress, and modulation of regulated cell death, which positions metformin as a multifaceted cardioprotective agent. However, inconsistent clinical outcomes warrant further research to define its role, particularly in non-diabetic populations.

### 3.2 Metformin and heart failure

Heart failure (HF) is a clinical syndrome characterized by symptoms and/or signs resulting from structural or functional cardiac abnormalities, confirmed by elevated natriuretic peptide levels or objective evidence of pulmonary or systemic congestion (Bozkurt et al., 2021). HF remains a leading global cause of morbidity and mortality, driven by reduced cardiac output and progressive ventricular remodeling that compromise quality of life and survival. Increasing evidence suggests that metformin may serve as a promising therapeutic option in HF.

#### 3.2.1 Preclinical evidence

Experimental studies have shown that metformin exerts cardioprotective effects through multiple complementary mechanisms. By activating AMPK, metformin enhances myocardial energy metabolism and mitigates oxidative stress, thereby improving cellular resilience under stress conditions (Dutta et al., 2023; Gundewar et al., 2009; Salvatore et al., 2021; Li et al., 2020; Loi et al., 2021; Chen et al., 2021). In LPS-induced septic myocardial injury, metformin reverses metabolic disturbances, improves survival and cardiac function, and reduces inflammation, apoptosis, and oxidative stress (Li et al., 2025). Metabolomic analyses further implicate PI3K/AKT and MMP signaling as key mediators, supporting its therapeutic repurposing potential (Li et al., 2025).

#### 3.2.2 Clinical evidence

Clinical evidence has increasingly established metformin as safe and potentially beneficial in heart failure (HF). Although its use was previously discouraged because of concerns about lactic acidosis, contemporary data indicate that this risk is minimal with appropriate renal and hepatic monitoring (Rahman and Tuba, 2022; Inzucchi et al., 2015; Dziubak et al., 2018). In small clinical trials, metformin improved antioxidant capacity and left ventricular remodeling in heart failure patients without diabetes (Kamel et al., 2024). Large cohort studies demonstrate the safety of metformin and even suggest survival benefits in patients with moderate renal impairment or advanced HF (Inzucchi et al., 2015; Masoudi et al., 2005; Larsen et al., 2020; Lazarus et al., 2018). Moreover, observational studies and meta-analyses consistently associate metformin use in patients with type 2 diabetes and HF with significant reductions in major adverse cardiovascular events (MACE), HF-related hospitalizations, cardiovascular mortality, and all-cause mortality (Masoudi et al., 2005; Aguilar et al., 2011; Crowley et al., 2017; Eurich et al., 2007; Huang and Zhao, 2025; Bahardoust et al., 2024). Randomized trials further show that 6 months of metformin therapy reduces oxidative stress, attenuates left ventricular hypertrophy and stiffening, and preserves cardiac function (Dutta et al., 2023; Kamel et al., 2024). Notably, benefits extend to non-diabetic HF patients, where metformin enhances antioxidant capacity and prevents increases in left ventricular mass (Kamel et al., 2024).

#### 3.2.3 Mechanistic insights

Metformin’s cardioprotective effects are mediated through several interrelated mechanisms. Activation of AMPK enhances myocardial energy efficiency and reduces oxidative stress, while anti-remodeling actions limit ventricular hypertrophy and fibrosis (Dutta et al., 2023; Gundewar et al., 2009; Salvatore et al., 2021; Li et al., 2020; Loi et al., 2021; Chen et al., 2021). Through modulation of PI3K/AKT and MMP signaling, metformin exerts anti-inflammatory and anti-apoptotic effects that preserve cardiac structure and function (Li et al., 2025; Ashayeri Ahmadabad et al., 2025). Additionally, its ability to correct disturbances in key metabolic pathways enhances overall cardiac resilience under stress (Osorio-Llanes et al., 2023).

#### 3.2.4 Summary

Metformin is a safe and effective adjunct in HF management, particularly for patients with type 2 diabetes, and shows promise in selected non-diabetic HF populations. Its benefits extend beyond glycemic control, encompassing improved myocardial energetics, reduced oxidative stress, and attenuation of adverse remodeling. These findings support further investigation into metformin’s role as a cardioprotective agent in HF.

### 3.3 Metformin and diabetic cardiomyopathy

Diabetic cardiomyopathy (DCM) is a distinct cardiac condition characterized by structural and functional myocardial abnormalities in diabetic patients, independent of coronary artery disease or hypertension (Zhao et al., 2022). It manifests as impaired contractility, ventricular hypertrophy, and myocardial fibrosis, often progressing to heart failure. With the global rise in diabetes prevalence, interest in metformin’s cardioprotective mechanisms in DCM has intensified.

#### 3.3.1 Preclinical evidence

Preclinical studies demonstrate that metformin protects against diabetic cardiomyopathy (DCM) through multiple complementary mechanisms (Dawood et al., 2022; Cameron et al., 2016; Zhao et al., 2021; Liu et al., 2022). In animal and cellular models, metformin reduces cardiomyocyte hypertrophy and lactate dehydrogenase release by downregulating HIF-1α and PPAR-γ, implicating the HIF-1α/PPAR-γ axis in limiting pathological growth (Liu et al., 2022). It also preserves myocardial structure by preventing desmin degradation and attenuating fibrosis via inhibition of the iNOS/mTOR/TIMP-1 pathway, thereby reducing collagen deposition and normalizing cardiac electrical activity (Dawood et al., 2022). Additionally, metformin activates the PK2/PKR–AKT/GSK3β signaling cascade, enhancing cardiomyocyte survival through Bcl-2 upregulation and reducing hyperglycemia-induced apoptosis and fibrosis (Yang et al., 2020; Bu et al., 2022). These findings underscore metformin’s capacity to mitigate structural remodeling and improve myocardial resilience in experimental DCM.

#### 3.3.2 Clinical evidence

Clinical studies support a cardioprotective role for metformin in diabetic cardiomyopathy and related cardiac complications. In the landmark *UK Prospective Diabetes Study (UKPDS)*, metformin use in overweight patients with type 2 diabetes significantly reduced diabetes-related endpoints, myocardial infarction, and all-cause mortality compared with conventional therapy, effects that persisted on long-term follow-up (Anonymous, 1998). Subsequent meta-analyses and large observational cohorts have consistently shown that metformin use is associated with lower all-cause and cardiovascular mortality and reduced heart failure (HF) incidence in diabetic populations (Crowley et al., 2017; Lamanna et al., 2011; Han et al., 2019). In patients with established HF, metformin therapy has been linked to improved survival and fewer hospitalizations compared with other glucose-lowering agents, supporting its safety and potential benefit even in systolic dysfunction (Aguilar et al., 2011; Eurich et al., 2013). Extending these benefits to earlier stages of metabolic disease and aging, a recent clinical study reported that metformin reduced the risk of frailty progression in prefrail older adults with hypertension and prediabetes, a population at high risk for cardiovascular dysfunction (Santulli et al., 2024). Moreover, small prospective trials demonstrated that metformin reduces left ventricular (LV) mass and improves diastolic function and myocardial energetics, suggesting favorable effects on cardiac remodeling central to DCM pathophysiology (Mohan et al., 2019). Collectively, these findings indicate that metformin confers both metabolic and direct myocardial benefits, although large randomized trials specifically targeting DCM outcomes are still needed.

#### 3.3.3 Mechanistic insight

Mechanistically, metformin exerts cardioprotection through coordinated regulation of inflammation, metabolism, and oxidative stress. It suppresses IL-6, TNF-α, and CRP while upregulating IL-10, thereby reducing myocardial inflammation (Almohaimeed et al., 2025; Yang et al., 2019; Schulz et al., 2024). Activation of AMPK with concurrent mTOR inhibition dampens NLRP3 inflammasome activity, limits IL-1β–mediated pyroptosis, and restores autophagic flux, effects abolished when AMPK is inhibited (Yang et al., 2019; Ghazal et al., 2025). Metformin further enhances oxidative stress resistance via upregulation of Klotho and GDF-15, promoting mitochondrial efficiency and anti-senescence signaling (Zhang et al., 2012). By improving mitochondrial function and indirectly activating Nrf2, metformin augments antioxidant capacity and contributes to sustained cardiometabolic protection (Mthembu et al., 2025).

#### 3.3.4 Summary

Metformin demonstrates multifaceted cardioprotective effects in DCM, targeting hypertrophy, fibrosis, inflammation, oxidative stress, and apoptosis through interconnected signaling pathways. These benefits, largely mediated by AMPK activation and modulation of HIF-1α/PPAR-γ, PK2/PKR–AKT/GSK3β, and mTOR/NLRP3 axes, position metformin as a promising therapeutic strategy for preventing and managing DCM. Further clinical trials are warranted to confirm these findings and define its role in routine care.

### 3.4 Metformin and doxorubicin-induced cardiotoxicity

Doxorubicin (DOX), a potent anthracycline chemotherapeutic, is widely used in oncology but its clinical utility is limited by dose-dependent cardiotoxicity. The cardiac injury arises from multiple interrelated mechanisms, including oxidative stress, mitochondrial dysfunction, apoptosis, inflammation, iron accumulation, topoisomerase IIβ inhibition, and dysfunctional autophagy/mitophagy (Zhang et al., 2012; Tadokoro et al., 2020; Singh et al., 2022; Bhutani et al., 2025). Metformin has recently gained attention as a potential cardioprotective agent against DOX-induced cardiac injury. Emerging evidence from preclinical and translational studies suggests that metformin attenuates myocardial damage, preserves cardiac function, and may also enhance DOX’s anticancer efficacy (Singh et al., 2022; Osataphan et al., 2023; Huang et al., 2025; Maghraby et al., 2025; El-Rayes et al., 2023).

#### 3.4.1 Preclinical evidence

Extensive preclinical research supports metformin’s cardioprotective role in DOX-induced cardiotoxicity (Singh et al., 2022; Zilinyi et al., 2018; Kobashigawa et al., 2014; Asensio-Lopez et al., 2011; Ajzashokouhi et al., 2019; Satyam et al., 2023; Sun et al., 2024a; Sun et al., 2024b). In rodent models, metformin markedly reduces serum markers of myocardial injury, limits cardiac fibrosis, and preserves left-ventricular function (Maghraby et al., 2025; Satyam et al., 2023; Sun et al., 2024a). Meta-analyses and animal studies confirm these benefits, highlighting consistent reductions in oxidative damage, apoptosis, and mitochondrial injury (Maghraby et al., 2025; Kobashigawa et al., 2014; Asensio-Lopez et al., 2011; Sun et al., 2024a). Metformin has been shown to lower ROS generation, restore mitochondrial membrane potential, and enhance antioxidant defenses through increased glutathione content and reduced malondialdehyde levels, collectively preserving mitochondrial integrity and cardiac function (Maghraby et al., 2025; Zilinyi et al., 2018; Argun et al., 2016). Novel formulations such as mitochondria-targeted metformin nanoparticles further improve mitochondrial morphology, calcium handling, and redox balance without diminishing DOX’s anticancer efficacy (Huang et al., 2025). Together, these findings underscore metformin’s potential to prevent structural and functional deterioration associated with anthracycline cardiotoxicity.

#### 3.4.2 Clinical evidence

Despite strong preclinical evidence, clinical data on metformin’s cardioprotective effects remain limited and somewhat inconsistent. Small randomized and observational studies in cancer patients have yielded mixed results. A phase II pilot trial (NCT02472353) designed to assess metformin’s effect on LVEF in breast cancer patients receiving DOX was terminated early due to low accrual. Similarly, the addition of metformin to standard breast cancer therapy did not significantly improve invasive disease-free survival (Goodwin et al., 2022). Although one randomized trial found no measurable change in cardiac function, metformin preserved mitochondrial respiration in peripheral blood mononuclear cells, suggesting a subclinical benefit (Osataphan et al., 2023). Large cohort data from US cancer survivors indicate that metformin use correlates with a reduced risk of cardiometabolic disease and cardiovascular mortality (Li et al., 2024), and another clinical study found lower rates of heart failure and mortality among patients receiving both anthracyclines and metformin (Onoue et al., 2023). Collectively, these findings are encouraging but highlight the need for large-scale, double-blind prospective trials to definitively establish metformin’s cardioprotective efficacy in oncology populations exposed to DOX.

#### 3.4.3 Mechanistic insight

Metformin mitigates DOX-induced cardiotoxicity through both AMPK-dependent and -independent mechanisms. AMPK activation contributes to suppression of ROS generation, preservation of mitochondrial bioenergetics (Yang et al., 2020), and normalization of autophagy (Zilinyi et al., 2018). Metformin promotes mitochondrial quality control via AMPK-driven mitochondrial biogenesis (Wu, 2023; Emelyanova et al., 2021), enhances PINK1/Parkin-mediated mitophagy, and prevents mitochondrial fragmentation by upregulating fusion proteins MFN1 and MFN2(115). These processes restore ATP production, maintain calcium homeostasis, and stabilize mitochondrial architecture (Maghraby et al., 2025). In parallel, metformin suppresses apoptosis by reducing the Bax/Bcl-2 ratio and caspase-3 activation (Van et al., 2023; Chen et al., 2020) and inhibits pro-inflammatory signaling through NF-κB and cytokines such as TNF-α and IL-6 (Malaekeh-Nikouei et al., 2023; Feng et al., 2021; Soraya et al., 2012). Anti-inflammatory and antioxidant actions also contribute to its synergistic anticancer effects by dampening STAT3-driven tumor-promoting feedback and inhibiting oncogenic pathways including mTOR, PI3K/Akt, and Wnt/β-catenin (Jalali et al., 2024; Hirsch et al., 2013; Li et al., 2018; Amable et al., 2019). However, the role of AMPK is complex. While AMPK activation by metformin generally confers cardioprotection, some studies report that deletion of the AMPKα2 isoform can actually mitigate DOX-induced injury (Pozzo et al., 2019), suggesting that different AMPK isoforms may have distinct or even opposing functions. Moreover, the contribution of mitophagy to DOX cardiotoxicity remains debated. Metformin has been shown to inhibit autophagy and mitophagy (Huang et al., 2020; Van et al., 2023), reducing DOX-induced cardiomyocyte death. Thus, metformin’s cardioprotective (Zilinyi et al., 2018; Kobashigawa et al., 2014; Asensio-Lopez et al., 2011; Ajzashokouhi et al., 2019) and antitumor effects (Peng et al., 2017; El-Ashmawy et al., 2017; Viollet et al., 2012; Cho et al., 2013) likely arise from an integrated network of AMPK-dependent and alternative pathways, warranting further isoform-specific investigation.

#### 3.4.4 Summary

Preclinical studies consistently demonstrate that metformin attenuates DOX-induced cardiotoxicity through coordinated modulation of oxidative stress, mitochondrial dynamics, autophagy, apoptosis, and inflammation. These effects translate into preserved cardiac structure, function, and energetic efficiency in animal models. However, clinical translation remains uncertain due to inconsistent trial outcomes and unresolved mechanistic questions, particularly regarding AMPK isoform-specific roles. Methodological variability among studies, including differences in dosing regimens, timing, and cardiac endpoints, further limits cross-comparison. Future research should emphasize standardized experimental protocols, mechanistic clarification of AMPK signaling, and large-scale randomized trials to establish whether metformin can reliably protect the human heart during anthracycline chemotherapy.

## 4 Dose-response and tissue-specific effects of metformin-mediated cardioprotection

The cardioprotective efficacy of metformin is governed by its unique pharmacokinetic profile and a dose-dependent bifurcation in molecular signaling. As a hydrophilic base, metformin relies on specialized transporters, namely, Organic Cation Transporters (OCT1-3), for cellular entry. While OCT1 is predominantly hepatic, the high expression of OCT3 in the myocardium facilitates tissue penetration and accumulation in cardiomyocytes (Chen et al., 2010; Solbach et al., 2011). Once inside the cell, metformin’s positive charge leads to its sequestration within the negatively charged mitochondrial matrix, where it can reach concentrations up to 1,000-fold higher than in the extracellular space (Bridges et al., 2016). This extensive volume of distribution and lack of plasma protein binding ensure that the heart is a primary site for its pleiotropic effects, though its renal excretion necessitates careful monitoring in heart failure patients to avoid toxicity (Graham et al., 2011).

The cardioprotective effects of metformin are both dose-dependent and tissue-specific (Table 2). At low doses (≈125 μg/kg), metformin activates AMPK, enhancing endothelial nitric oxide synthase (eNOS) activity, improving vascular function, and reducing myocardial injury (Calvert et al., 2008). Similar doses improve left ventricular function and survival in heart failure models (Gundewar et al., 2009). These protective effects occur at concentrations far below those needed for glycemic control, indicating a distinct cardiac mechanism (Zhou et al., 2001). In contrast, higher concentrations inhibit mitochondrial complex I, thereby reducing oxidative stress and delaying the opening of the mitochondrial permeability transition pore (mPTP), a key event in ischemia-reperfusion injury (Fontaine, 2018; Zilov et al., 2019). Thus, metformin’s cardiac actions depend on its local concentration. Lower levels favor AMPK signaling, whereas higher levels exert direct mitochondrial effects. Understanding this dual mechanism underscores the need for dose optimization and precise pharmacokinetic profiling to enhance metformin’s therapeutic potential while minimizing adverse effects in cardiovascular disease management.

**TABLE 2:** Metformin dose & cardioprotection (selected studies).

Study	Model/Species	Dose & route	Timing/Duration	Cardioprotection endpoints	Mitochondrial/ROS mechanism
Calvert et al. (2008)	Mouse I/R	125–250 μg/kg i.v	Before ischemia or at reperfusion (acute)	↓Infarct size, improved function	AMPK activation, eNOS phosphorylation; mitochondrial protection
Solskov et al. (2008)	Rat (Langendorff)	250 mg/kg oral	Single dose, 24h later	Protection vs. ischemia *ex vivo*	AMPK activation; mitochondrial energetics
Kanamori et al. (2019)	Mouse DCM (δ-sarcoglycan–def.)	200 mg/kg/day (pump)	4 weeks (chronic)	Improved LV function, ↓fibrosis	Enhanced autophagy (↑LC3-II)
Cittadini et al. (2012)	Rat chronic HF (SHHF)	100 mg/kg/day (drinking water)	12 months	Prevented CHF development	Improved energetics, ↓fibrosis
Techiryan et al. (2018)	Swine MI	5 mg/kg IV + 1 mg/kg/min infusion	At reperfusion	No infarct size reduction	No mitochondrial benefit
Emelyanova et al. (2021)	Human iPSC-CMs	≤2.5 mM vs. ≥ 5 mM	Acute *in vitro*	Low conc ↑OCR; high conc ↓OCR	Biphasic mitochondrial effects via AMPK/complex I
Bates et al. (2023)	Aged mouse I/R	High dose (reported acute)	At reperfusion	↓Infarct size, improved recovery	Mitochondrial protection in aged heart
Argun et al. (2016)	Rat DOX cardiotoxicity	50 & 500 mg/kg oral	Concurrent with DOX	↓Cardiotoxicity markers	↓ROS, improved mitochondrial function
Sun et al. (2024b)	H9C2 & mouse DOX	Various; ∼2.9 mg/kg *in vivo*	Acute & chronic	Dose-dependent protection	Mitophagy and mitochondrial dynamics
Mohan et al. (2019) (MET-REMODEL)	Human CAD/LVH (non-diabetic)	2000 mg/day oral	12 months	↓LV mass, improved LVEF	Metabolic/Mitochondrial remodeling
Kanamori et al. (2019), Xiang et al. (2024), Yang et al. (2019) (rodent diabetic CM)	Mouse/Rat diabetic CM	100–300 mg/kg/day	Weeks–months	↑LV function, ↓fibrosis	↓ROS, improved mitochondria & mitophagy

## 5 Metformin’s safety profile and clinical considerations

Metformin is generally well tolerated, with gastrointestinal symptoms (nausea, diarrhea, metallic taste, flatulence) being the most common adverse effects; these can be minimized by taking the drug with meals (F and roldi, 2024). Long-term use may also reduce vitamin B12 absorption, warranting periodic monitoring during extended therapy (Dutta et al., 2023; Top et al., 2022). In addition to these well-recognized effects, emerging experimental evidence suggests that metformin may influence systemic metabolism beyond classical peripheral targets. For example, combined administration of metformin and the thiamine antagonist amprolium altered free amino acid metabolism in the rat brain, leading to changes in behavior and heart rate, highlighting potential central and autonomic effects under conditions of metabolic stress or drug interaction (Graf et al., 2024). Given its expanding role in cardiometabolic disease, renal function and heart failure status must guide clinical use. Metformin is contraindicated at eGFR <30 mL/min/1.73 m^2^ and should be dose-adjusted or avoided when eGFR is 30–44 mL/min (Lipska et al., 2011; Salpeter et al., 2010). Although once avoided in congestive heart failure, current evidence supports its safety and potential benefit in stable CHF, with discontinuation advised during acute decompensation (Aguilar et al., 2011; Eurich et al., 2007; Benes et al., 2022; Wang et al., 2021; Eurich et al., 2009).

The most serious but rare complication is metformin-associated lactic acidosis (MALA), typically occurring in the setting of renal impairment and mechanistically linked to mitochondrial complex I inhibition (Dutta et al., 2023; Graham et al., 2011; Zhou et al., 2001). Although exceedingly uncommon (∼0.03 cases per 1,000 patient-years), MALA carries a high mortality rate (∼50%) (Benes et al., 2022; Wang et al., 2021). It most often develops with renal or hepatic failure, severe heart failure, sepsis, or excessive alcohol use, underscoring the need for close monitoring and immediate discontinuation if signs of metabolic acidosis emerge (Bailey and Turner, 1996; Stades et al., 2004; Lalau and Race, 2001). Because conventional formulations are limited by poor bioavailability, gastrointestinal intolerance, and suboptimal tissue targeting, innovative drug delivery systems, such as nanoparticles, pH-responsive hydrogels, and microneedles, are being developed to enhance therapeutic efficacy, reduce adverse effects, and improve patient adherence (Sulong et al., 2025). For example, metformin-loaded nanoparticles attenuate hyperglycemia-associated oxidative stress and promote eNOS phosphorylation in vascular endothelial cells (Mohamed et al., 2024), supporting the feasibility of nanoparticle-based drug delivery strategies.

## 6 Summary and future perspectives

Metformin, traditionally a first-line therapy for type 2 diabetes, is increasingly recognized as a pleiotropic cardiometabolic agent. Its cardiovascular benefits arise through dose- and tissue-dependent mechanisms: low concentrations predominantly activate AMPK to enhance endothelial function and reduce myocardial injury (Calvert et al., 2008), while higher concentrations inhibit mitochondrial complex I to limit oxidative stress and delay mPTP opening (Fontaine, 2018; Zilov et al., 2019). These mechanistic distinctions highlight the need for optimized dosing and improved pharmacokinetic understanding. Clinically, metformin is well tolerated, with gastrointestinal symptoms and vitamin B12 deficiency representing the most common adverse effects (F and roldi, 2024; Top et al., 2022). The rare occurrence of metformin-associated lactic acidosis underscores the importance of renal function-guided prescriptions (Lipska et al., 2011; Salpeter et al., 2010).

Across cardiovascular contexts, metformin demonstrates broad protective effects (Table 3). It attenuates ischemia/reperfusion injury by suppressing NLRP3 inflammasome and apoptosis, mitigating ferroptosis via Nur77-IDH1 signaling (Zhang et al., 2020; Stone et al., 2024; Wu et al., 2025), and mimicking ischemic preconditioning (Rozier et al., 2021). In heart failure and diabetic cardiomyopathy, metformin reduces oxidative stress, fibrosis, and adverse remodeling through coordinated AMPK-dependent and independent pathways (Kamel et al., 2024; Liu et al., 2022; Yang et al., 2020). Metformin also shows promise against doxorubicin-induced cardiotoxicity, though variability in preclinical models calls for standardized approaches (Singh et al., 2022; Zilinyi et al., 2018; Kobashigawa et al., 2014; Asensio-Lopez et al., 2011; Ajzashokouhi et al., 2019; Satyam et al., 2023; Sun et al., 2024a; Sun et al., 2024b).

**TABLE 3:** Cardioprotective and Therapeutic Effects of Metformin Across cardiac pathologies.

Population/Disease	Key effects & evidence	Proposed mechanisms
Non-diabetic (general)	Weight loss, improved lipid/insulin profile, some anti-atherosclerotic effects (e.g., CAMERA trial), mild improvements in cardiac function (Preiss et al., 2014)	AMPK, PEN2-lysosome signaling (Ma et al., 2022), anti-inflammatory, insulin modulation
Cardiovascular disease	Slows atherosclerosis progression; reduces vascular thickening; improves myocardial ischemia (in some non-diabetic trials); reduces MI risk in diabetics (Holman et al., 2008)	Energetic remodeling, endothelial effects, anti-inflammation
Heart failure	Reduced all-cause mortality in diabetics with HF; improves LV hypertrophy, oxidative stress, and systolic/diastolic function; cautious use due to lactic acidosis risks (Kamel et al., 2024; Salvatore et al., 2021)	AMPK activation, improved bioenergetics, anti-fibrosis mechanisms
Diabetic cardiomyopathy	↓ heart weight/body weight ratio, suppresses hypertrophy, preserves structure, modulates fibrosis and anti-apoptotic pathways	PK2/PKR/AKT/GSK3β axis, iNOS/mTOR/TIMP-1 axis, AMPK-dependent/independent mechanisms (Dawood et al., 2022; Yang et al., 2020; Bu et al., 2022)
Ischemia-reperfusion injury	↓ NLRP3 inflammasome, ↓apoptosis & ferroptosis, ↓ infarct size; mimics ischemic preconditioning (Zhang et al., 2020; Rozier et al., 2021; Stone et al., 2024)	Activates Akt, ↑ Bcl-xL, reduces ROS (Rozier et al., 2021; Stone et al., 2024), ↑ Nur77-IDH1 (57)
Chemotherapy-induced cardiotoxicity (e.g., Doxorubicin)	Mitigates structural/functional damage (preclinical); effect on autophagy/mitophagy is controversial	PINK1/Parkin pathways, mitochondrial preservation (Ma et al., 2023), AMPK (complex role)
Limitation/Uncertainties	Mixed results in non-diabetic CVD and oncology trials, context-dependent autophagy/AMPK effects, need for more targeted studies	​

Despite strong evidence in diabetic populations, results in non-diabetic individuals remain inconsistent. Trials such as CAMERA and GIPS-III suggest slowed atherosclerosis progression or improved post–myocardial infarction remodeling, whereas others show no clear cardiovascular benefit. As summarized in Table 4, preclinical and clinical studies support cardioprotective effects of metformin across diverse cardiac conditions, but large, rigorously designed trials are needed to define its role beyond metabolic indications. Ongoing studies, including VA-IMPACT (NCT02915198) for secondary prevention in patients with prediabetes and atherosclerotic cardiovascular disease and emerging trials in atrial fibrillation (e.g., NCT05878535), will be critical. If consistent benefits are confirmed, metformin could be considered as adjunct therapy for high-risk non-diabetic patients; until then, its use should remain confined to established metabolic indications.

**TABLE 4:** Metformin’s cardioprotective effects: preclinical and clinical evidence.

Condition	Preclinical evidence	Clinical evidence	Strength of evidence
Ischemia-reperfusion injury	Robust animal studies show ↓ infarct size, improved LV remodeling via AMPK-eNOS activation, mitochondrial protection, anti-oxidative effects	Beneficial in diabetic patients (Mone et al., 2025; Holman et al., 2008; Bromage et al., 2019); inconsistent in nondiabetic patients: GIPS-III trial (Lexis et al., 2014; Hartman et al., 2017) and Camera trial (Preiss et al., 2016; Preiss et al., 2014)	Low to moderate
Heart failure	Animal models: improved LV function, ↓ oxidative stress, ↑ AMPK signaling	NCT05177588- Metformin add-on in non-diabetic HFrEF; meta-analysis of 15 RCTs in T2DM + CHF shows mortality benefit (RR ≈ 0.72)	Low to moderate
Diabetic cardiomyopathy	Strong mechanistic rationale; animal studies show improved myocardial structure/function, ↓ fibrosis, ↑ mitochondrial efficiency	No large RCTs; ongoing observational studies (e.g., NCT05958706, NCT05915260)	Moderate
Doxorubicin cardiotoxicity	Consistent protective effects in animal models (↓ oxidative stress, apoptosis, improved mitochondrial dynamics); meta-analysis confirms reliability	NCT02472353- Pilot RCT in breast cancer patients; RCT in 143 patients during DOX therapy showed mitochondrial protection but no significant troponin/LVEF benefit	Moderate

Looking ahead, several research priorities will shape the future of metformin in cardiovascular medicine. First, clarifying the isoform-specific roles of AMPK, particularly AMPKα1 versus AMPKα2 in distinct cardiac cell types, is essential for understanding the context-dependent nature of metformin’s effects. Tissue-specific genetic models, single-cell transcriptomics, and proteomic approaches will be critical for defining these pathways. Equally important is expanding investigation into alternative AMPK activation mechanisms, such as the PEN2-dependent lysosomal pathway, which may offer safer therapeutic avenues for patients at risk of mitochondrial dysfunction or lactic acidosis.

Secondly, metformin’s context-dependent regulation of autophagy and mitophagy remains an area of notable uncertainty. Divergent findings across studies reflect variations in dosing, model systems, disease context, and timing. Advanced tools, including *in vivo* autophagy/mitophagy reporters, high-resolution mitochondrial imaging, and integrative multi-omics, are needed to resolve these discrepancies and identify key downstream mediators such as NLRP3, the Nur77-IDH1 axis, and regulators of ferroptosis and pyroptosis.

Finally, success in clinical translation will depend on innovative delivery strategies and refined dosing paradigms (Sulong et al., 2025). Cardiac-targeted nanoparticles, low-dose pulses, or intermittent regimens could enhance bioavailability and efficacy while reducing systemic exposure and toxicity. Standardization of preclinical models, especially for doxorubicin cardiotoxicity, will be essential for reconciling inconsistencies in proposed mechanisms.

In summary, metformin’s expanding cardiometabolic profile suggests potential to reshape cardiovascular disease management. Beyond glucose lowering, metformin exerts pleiotropic effects that include endothelial protection, preservation of mitochondrial function, modulation of redox homeostasis, suppression of inflammation, attenuation of fibrosis, and regulation of autophagy (Table 1). These actions confer metabolic and cardioprotective benefits in both diabetic and non-diabetic populations and support its candidacy as a broad-spectrum cardioprotective agent. However, despite strong mechanistic and translational evidence, clinical outcomes in non-diabetic cohorts remain inconsistent. Ongoing trials, together with large, well-controlled studies, will be essential to determine whether these mechanistic benefits translate into meaningful cardiovascular risk reduction across diverse patient populations.

## Glossary

T2DM: type 2 diabetes mellitus
AMPK: AMP-activated protein kinase
LKB1: liver kinase B1
ETC: electron transport chain
mTOR: mammalian of mechanistic target of rapamycin
TFEB: Transcription Factor EB
eNOS: endothelial nitric oxide synthase
PEN2: presenilin enhancer 2
mGPD2: mitochondrial glycerol-3-phosphate dehydrogenase
G3P: glycerol-3-phosphate
DHAP: dihydroxyacetone phosphate
ULK: unc-51 like autophagy activating kinase
LV: left ventricle
LVEF: Left Ventricular Ejection Fraction
CAD: coronary artery disease
AMI: acute myocardial infarction
STEMI: ST-Elevation Myocardial Infarction
I/R: ischemia-reperfusion
NLRP3: NLR Family Pyrin Domain Containing 3
ROS: reactive oxygen species
mPTP: mitochondrial permeability transition pore
LPS: Lipopolysaccharide
MMP: matrix metalloproteinase
TIMP-1: Tissue Inhibitor of Metalloproteinases-1
HF: Heart failure
DCM: Diabetic cardiomyopathy
LDH: lactate dehydrogenase
PPARγ: peroxisome-proliferator-activated receptor γ
PGC-1α: PPARγ co-activator-1α
GDF-15: growth differentiation factor 15
PK2: Prokineticin 2
PKR1/2: prokineticin receptor1/2
GSK3B: Glycogen synthase kinase-3 beta
TNF-α: Tumor Necrosis Factor alpha
CRP: C-reactive protein
DOX: doxorubicin
IL-6: Interleukin 6
PINK1: phosphatase and tensin homolog (PTEN)–induced putative kinase 1
Mfn1/2: Mitofusins1/2
GLP-1: glucagon-like peptide-1
SGLT1: sodium-glucose cotransporter 1
RCT: Randomized Controlled Trial
